# Epidemiology, Treatment Patterns, Survival, Healthcare Resource Utilization, and Costs of Dedifferentiated Liposarcoma (DDLPS) in Canada: A Retrospective Cohort Study Using Administrative Databases in Ontario

**DOI:** 10.3390/curroncol32050273

**Published:** 2025-05-09

**Authors:** Soo Jin Seung, Anisia Wong, Raymond Milan, Nisha Chandran, Albiruni R. Abdul Razak

**Affiliations:** 1 HOPE Research Centre, Sunnybrook Research Institute, Toronto, ON M4N 3M5, Canada; anisia.wong@sri.utoronto.ca; 2 Boehringer Ingelheim Canada Ltd., Burlington, ON L7L 5H4, Canada; raymond.milan@boehringer-ingelheim.com (R.M.); nisha.chandran@boehringer-ingelheim.com (N.C.); 3 Toronto Sarcoma Program, Princess Margaret Cancer Centre, University Health Network, Toronto, ON M5G 2M9, Canada; albiruni.razak@uhn.ca

**Keywords:** dedifferentiated liposarcoma, treatment patterns, survival, healthcare resource utilization, costs

## Abstract

Background: Dedifferentiated liposarcoma (DDLPS) is a rare, aggressive tumour with poor survival outcomes in advanced settings. This study assessed the incidence/prevalence, treatment patterns, survival, healthcare resource utilization (HCRU), and costs for DDLPS patients in Ontario, Canada. Methods: A retrospective cohort study was conducted among DDLPS patients between 2010 and 2022 using administrative databases. Overall survival, all-cause HCRU, and costs (2023 Canadian dollars, CAD) were compared based on advanced disease and resection status. Results: The overall incidence and cumulative prevalence of DDLPS was 0.465 and 1.995 per 100,000 people, respectively. Of all 611 DDLPS cases (64.3% male, median age [IQR]: 67 [57–76] years), 40.3% and 61.0% had advanced and unresected disease, respectively. The median overall survival (mOS) was 69 months [IQR = 15–151] for the entire cohort, but this was significantly lower for advanced and unresected disease (*p* < 0.0001). Among patients receiving systemic treatments (N = 117), 81.2% were prescribed doxorubicin as first-line treatment. All-cause healthcare costs (2023 CAD) amounted to CAD 34,448 per person-year (PPY), with inpatient hospitalizations being the highest cost driver at CAD 14,522 PPY and 0.8 inpatient hospitalization PPY for all years. Advanced disease had higher HCRU and costs. Conclusions: This is the first comprehensive real-world evidence study that quantifies the high mortality and cost burden associated with DDLPS in Canada.

## 1. Introduction and Objectives

Liposarcoma (LPS) is a rare mesenchymal malignancy of adipose tissue, accounting for approximately 15–20% of all soft tissue sarcoma (STS) cases [1,2,3,4]. LPS can be further classified into five subtypes as follows: well-differentiated liposarcoma (WDLPS), dedifferentiated liposarcoma (DDLPS), myxoid liposarcoma (MLPS), pleomorphic liposarcoma (PLPS) [2], and myxoid pleomorphic liposarcoma (MPLPS) [5]. Among these, DDLPS comprises 14.3% of all LPS [6].

DDLPS is highly aggressive, characterized by a loco-regional recurrence rate of 40% and a metastatic rate of 15–30%, commonly affecting the liver and lungs [7]. Consequently, DDLPS has a poor prognosis, with a 5-year overall survival rate of 30–40% [6,8,9]. The largest study on DDLPS, conducted by Gootee et al. (2018) [3] in the United States with 3573 patients, reported a median overall survival (mOS) of 10.2 ± 1.9 months for stage IV patients.

Treatment for localized DDLPS is surgery, with or without radiotherapy. However, in patients with unresected or advanced (metastatic) DDLPS, anthracycline-based chemotherapy is the recommended first-line (1L) treatment [5]. Doxorubicin-based chemotherapy remains the usual 1L regimen, while later-line treatments include gemcitabine-based combinations and other chemotherapies such as dacarbazine, eribulin, and trabectedin [2,4,5].

There is a notable gap in the existing literature pertaining to DDLPS within the Canadian context, with limited information available regarding its incidence/prevalence, treatment patterns, and the economic burden of the disease for Canadian DDLPS patients.

Since variations in patient demographics and healthcare systems can influence disease management and outcomes, it is necessary understand the unique challenges and characteristics of DDLPS within the context of a publicly funded healthcare system like Canada’s. This study seeks to address current gaps in knowledge about DDLPS and provide valuable insights into the epidemiology, clinical, and economic aspects of DDLPS in the Canadian healthcare landscape, ultimately resulting in more effective patient care and treatment strategies.

The present study aimed to (1) estimate the incidence and prevalence of DDLPS in Canada between 2010 and 2022; (2) describe how patients are treated for DDLPS and the sequence of treatment received; (3) assess the 1-, 5- and 10-year survival probabilities and mOS in patients with DDLPS; and (4) assess HCRU and costs related to DDLPS. The last two objectives were also compared in patients with advanced (metastatic) disease and resected status.

## 2. Methodology

### 2.1. Study Design, Population, and Data Sources

A retrospective cohort study was conducted in Ontario using databases housed at the Institute of Clinical and Evaluative Sciences (ICES). For this study, data were available from 1 January 2010 until 31 December 2023. Patient data were accessed via provincial administrative databases housed at ICES, which collect real-world data through linked population-level provincial databases. Individual patients who met the study inclusion criteria were linked to ten different databases to retrieve treatment and outcome data. The Ontario Cancer Registry (OCR) provided cancer diagnosis data (ICD-0-3 derived from ICD-10 codes), while the Registered Persons Database (RPDB) offered demographic information such as age, sex, and date of death. Drug-specific databases, including the New Drug Funding Program (NDFP), Ontario Drug Benefit (ODB) Program, and Activity Level Reporting (ALR), supplied details on drug prescriptions and chemotherapies administered at cancer centres. The National Ambulatory Care Reporting System (NACRS) and Discharge Abstract Database (DAD) provided outpatient, inpatient, and procedural data. Physician visits (general practitioners and specialists) were accessed via the Ontario Health Insurance Plan (OHIP). Lastly, the Continuing Care Reporting System (CCRS) detailed long-term care, and the Home Care Database (HCD) captured home care service usage in Ontario.

### 2.2. Study Population and Follow-Up

Patients in Ontario, Canada, diagnosed with DDLPS between 1 January 2010 and 31 December 2022 were identified using the ICD-O-3 code 8858/3 in the OCR. Patients with certain topology or ICD-10 codes that were not related to DDLPS were excluded based on clinical judgement, and only those with ICD-10 codes of 48.x, 49.x, and 38.x were considered [10]. Pathology information was not available. Index date was defined as the date of first DDLPS diagnosis in the OCR. Patients were also included if they were Ontario residents aged 18 to 105 years with a valid ICES Key Number (IKN) and were eligible for OHIP. Lastly, to address objectives 2–4, patients with a history of previous cancers in the 5 years prior to index date were excluded, except for effectively treated non-melanoma skin cancers, carcinoma in situ of the cervix, and ductal carcinoma in situ. Patients were followed from index date until loss of OHIP eligibility, date of death, or 31 December 2023, using whichever date occurred first.

Patients were considered to have advanced DDLPS if they had stage III/IV at index date or if they initiated 1L systemic therapy during the follow-up; otherwise, patients were considered as not advanced. Patients were considered to have unresected DDLPS if they did not undergo any surgical intervention within six months of the index date; otherwise, DDLPS was considered as resected.

### 2.3. Outcomes

#### 2.3.1. Baseline Characteristics

Descriptive statistics were used to summarize the baseline characteristics, including age, sex, residence, income quintile, comorbidities, disease stage, topography, years of follow-up, and treatment type post-diagnosis.

#### 2.3.2. Incidence and Prevalence

Annual, overall incidence, and cumulative prevalence rates of DDLPS in Ontario were analyzed from 2010 to 2022. Data were obtained from the OCR for cancer cases and RPDB. Rates were calculated per 100,000 person-years using DDLPS cases (numerator) and the Ontario population (denominator). Cumulative prevalence included surviving DDLPS patients from prior years.

#### 2.3.3. Treatment Patterns

Treatment data were obtained from the three following databases: NDFP (IV chemotherapies only), ALR (IV chemotherapies and most oral therapies administered at cancer centres), and ODB (oral drugs for residents 65+ or on social assistance). Therapies were categorized into 1L and second-line (2L) treatments across three regimens as follows:

- DOXO: doxorubicin alone or with ifosfamide, cisplatin, or olaratumab.
- GEM: gemcitabine alone or with cisplatin or docetaxel.
- OTHER: not DOXO and GEM; includes carboplatin and paclitaxel with radiotherapy, dacarbazine, epirubicin, and ifosfamide; paclitaxel (weekly), pazopanib, vincristine, dactinomycin, and cyclophosphamide; ifosfamide, olaratumab (maintenance), paclitaxel, and carboplatin; and durvalumab.

The timeframe for treatment patterns was from the time of diagnosis until either the patient’s death or when other censoring variables occurred, whichever happened first. Changes within DOXO or GEM regimens did not count as a therapy change, while changes within OTHER regimens did.

#### 2.3.4. Overall Survival (OS)

OS was defined as the time of DDLPS diagnosis until either their date of death or the date of censoring. Probability of survival at 1, 5, and 10 years was also assessed.

#### 2.3.5. Healthcare Resource Utilization and Costs

All-cause healthcare resource utilization (HCRU) and all-cause healthcare costs were calculated using an administrative data-based macro [11]. The mean HCRU for each DDLPS patient per year (PPY) was measured from index date until the end of follow-up, with results for the full cohort, resected and unresected, and advanced and not advanced, as well as for all years and year 1. Cancer-related HCRU and costs (e.g., medical oncologist visits; cancer clinic visits) are highlighted in the main results. Other general HCRU and costs such as same-day surgery and oral medications were analyzed but not presented in the main results either due to low utilization or low costs.

### 2.4. Statistical Analyses

Statistical analyses were conducted in SAS Enterprise Guide 8.3. Study results included baseline characteristics and summary statistics, while counts and proportions were used to summarize categorical variables. Unadjusted and adjusted Cox regression models were used to estimate factors associated with survival among patients with DDLPS, calculating hazard ratios (HRs) for the overall cohort based on age (18–64 years vs. 65+ years), sex, tumour location (retroperitoneal/abdominal vs. combined other sites), and tumour stage, analyzed either as individual categories (I, II, III, IV) or grouped into early stage (I + II) versus advanced stage (III + IV). All-cause healthcare costs were reported as mean costs PPY. The mean cost PPY was calculated by dividing the total cost for all patients in the group by the total person-years for all patients in the group. The 95% confidence interval (CI) for the mean cost PPY was estimated using the bootstrapping method with 5000 resamples. All costs were expressed in 2023 Canadian dollars (2023 CAD) using the all-item consumer price index. OS, HCRU, and costs were also compared among DDLPS patients based on their advanced disease and resected status, and a *p*-value of <0.05 was assigned when comparator groups did not have an overlapping 95% CI.

## 3. Results

In total, 795 patients were diagnosed with DDLPS between 1 January 2010 and 31 December 2022. After the exclusion criteria, 684 patients were included in the incidence/prevalence analysis. After excluding patients with a history of cancer in the previous 5 years before the index date, 611 patients were considered for analyses related to survival outcomes, treatment patterns, all-cause HCRU, and costs (see Supplemental Figure S1).

### 3.1. Baseline Characteristics

Table 1 summarizes the baseline characteristics of the 611 DDLPS patients in Ontario. Most patients were male (64.3%), with 57.1% aged 65 or older, and 31.5% had 1–3 co-morbidities. Primary tumour sites were the retroperitoneum (60.6%) and extremities (19.8%). Stage III/IV cases accounted for 26.7% of diagnoses, and 48% had a missing or unknown disease stage. Of the 611 patients, 40.3% (N = 246) had advanced DDLPS based on a proxy measure of patients were either stage III or IV at diagnosis or received a systemic treatment during follow-up. A total of 61% of DDLPS patients were considered unresected since they did not receive a surgical resection within 6 months of diagnosis.

In general, the baseline characteristics were similar for the advanced (N = 246) and not advanced (N = 365) subgroups of patients. Advanced DDLPS patients had a mean age of 64.8 years compared to 67.1 years for not advanced patients. Retroperitoneal tumours were more common in advanced patients than not advanced patients (67.5% vs. 55.9%). Advanced patients were more likely to have received radiation therapy after DDLPS diagnosis (73.6%) compared to not advanced patients (59.5%).

Differences in baseline characteristics were found based on resected status. Resected patients (N = 238) had a mean age of 64.8 years, while unresected (N = 373) patients were older with a mean age of 67.0 years. Additionally, resected patients had a higher mean follow-up time (5.4 years) compared to unresected patients (3.3 years). Retroperitoneal tumours were more common in unresected patients (68.9%), while resected patients had a more even distribution between the retroperitoneum (47.5%) and extremities (39.5%). More resected patients were treated at primary cancer centres (63.4%) compared to unresected patients (44.5%). Unresected patients were more likely to receive radiation therapy (72.3%) and systemic therapy (22.8%) after DDLPS diagnosis compared to resected patients.

### 3.2. Incidence and Prevalence of DDLPS

The overall incidence and cumulative prevalence rates of DDLPS between 2010 and 2022 were 0.46 and 1.99 per 100,000, respectively (Table 2). Male patients and those of older age (65 years and older) had the highest incidence and prevalence rates of DDLPS (Table 2). Annual incidence and prevalence rates are reported in Supplemental Figure S2.

### 3.3. Treatment Patterns

Table 3 shows the types of chemotherapies received in 1L and 2L by regimen groups. Of the 611 patients with DDLPS, 117 (19.1%) received 1L systemic treatments. DOXO was the most prescribed as the 1L treatment (81.2%), followed by GEM (13.7%) and OTHER (5.1%). Among those treated with DOXO, 61.5% received this treatment as a monotherapy. Only 41 patients received 2L treatment (35% of 1L), with the majority receiving GEM (53.7%).

### 3.4. Survival Outcomes for Overall Cohort and Stratifications

**Figure 1:**
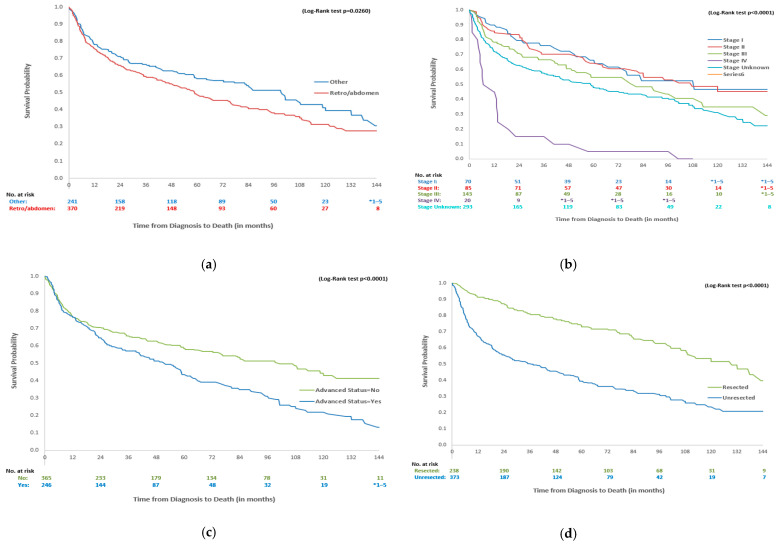
Survival curves for DDLPS patients by primary tumour site, disease stage, advanced status and unresected status. * 1–5 indicates small cell suppression as per ICES policy. (**a**) Overall survival of DDLPS patients by primary tumour site. (**b**) Overall survival of DDLPS patients by disease stage. (**c**) Overall survival of DDLPS patients by advanced status. (**d**) Overall survival of DDLPS patients by resected status.

Table 4 presents the survival probabilities at year 1, 5, and 10, and mOS of the overall DDLPS cohort as well as stratifications by age, disease stage, advanced disease, resected status, and line of treatment received. For all 611 DDLPS patients, mOS was 69 (IQR = 15–151) months, with 1-year, 5-year, and 10-year survival rates of 76.6%, 52.4%, and 35.0%, respectively. Patients with retroperitoneal or abdominal tumours had significantly lower mOS when compared to those with other tumour sites (60 [14–151] vs. 101 [17–NA] months, *p* < 0.0001, Figure 1a). Stage I patients had the best survival outcomes, with a 1-year survival of 90.0% and an mOS of 85 [IQR:43–NA] months. Conversely, stage IV patients had the lowest survival rates, with a 1-year survival of 45.0% and an mOS of 8 months (see Figure 1b). DDLPS patients with advanced disease had lower 1-year (76.4 months, IQR = 71.1–81.7), 5-year (43.7 months, IQR = 36.8–50.6), and 10-year (21.9 months, IQR = 14.7–29.1) survival rates than those without advanced status, with an mOS of 53 (IQR = 15–108) months, while it was 103 [IQR = 16–NA] months for non-advanced patients (*p* < 0.0001, Figure 1c). Lastly, more patients with resected DDLPS were alive at 1 year when compared to unresected DDLPS, with a 1-year survival rate of 91.2% and an mOS of 131 [IQR = 57–NA] months compared to a 1-year survival of 67.3% and an mOS of 38 [IQR = 9–115] months (*p* < 0.0001, Figure 1d). The mOS values for those receiving 1L (N = 117) and 2L (N = 41) treatments were 16 [IQR:6–58] and 9 [IQR:5–16] months, respectively.

According to the unadjusted and adjusted Cox regression models, older age (65+ years), retroperitoneum DDLPS, and stage IV disease were associated with a significantly increased risk of all-cause mortality (see Supplemental Table S1).

### 3.5. All-Cause Healthcare Resource Utilization (HCRU) and Costs

Table 5 reports the all-cause HCRU of the overall DDLPS cohort for all years and year 1 relevant to cancer care. For all years, specialist visits were used by all patients, with 22.1 (95% CI: 20.5–23.7) visits PPY. Among specialists, medical oncologist visits averaged 2.5 (95% CI: 1.9–3.1) PPY, and therapeutic radiologist visits were 1.8 (95% CI: 1.7–2.0) PPY. Inpatient hospitalizations averaged 0.8 (95% CI: 0.8–0.9) PPY, with a mean length of stay of 8.9 days (95% CI: 7.8–10.2). Homecare visits were utilized by over 80% of the overall cohort, with 19.0 (95% CI: 15.6–22.9) visits PPY while mean cancer clinic visits were 5.2 (95% CI: 4.8–5.6) PPY. All cancer-related HCRU values were substantially higher in year 1.

In Table 6, HCRU results were stratified by advanced and non-advanced status for all years, and *p*-values were determined. Specialist visits averaged 30.1 (95% CI: 27.2–33.2) PPY for advanced status compared to 17.7 (95% CI: 16.2–19.5) for non-advanced status, with medical oncologist visits significantly higher in advanced status patients, at 5.9 (95% CI: 4.5–7.4) PPY versus 0.6 (95% CI: 0.4–0.9) PPY in non-advanced status. Inpatient hospitalizations were also significantly higher for the advanced DDLPS cohort with 1.1 (95% CI: 1.0–1.3) hospitalization PPY compared to 0.7 (0.6–0.8) PPY for the non-advanced cohort. Lastly, cancer clinic visits were significantly higher for the advanced DDLPS cohort with 7.4 (95% CI: 6.6–8.4) visits PPY versus 4.0 (95% CI: 3.6–4.4) PPY for non-advanced patients. When stratified by resected and unresected status, no significant differences in HCRU were observed.

In Table 7, the total mean all-cause healthcare cost was CAD 34,448 (95% CI: CAD 31,394–CAD 37,863) PPY for all years and CAD 86,840 (95% CI: CAD 80,120–CAD 94,236) PPY for year 1 post-DDLPS diagnosis. It is important to note that these costs are not specific to DDLPS but represent the total healthcare costs of patients diagnosed with DDLPS. In terms of relevant cancer care for all years, inpatient hospitalizations accounted for the largest cost share at CAD 14,522 (95% CI: CAD 12,804–CAD 16,505) PPY, followed by specialist visits at CAD 4895 (95% CI: CAD 4564–CAD 5249), cancer clinic visits at CAD 4314 (95% CI: CAD 3860–CAD 4801), and homecare services at CAD 2122 (95% CI: CAD 1796–CAD 2486). The same cost trends were observed for year 1 but at higher cost values.

Table 8 presents all-cause healthcare costs results stratified by advanced and resected status. For all years, the total mean cost PPY was CAD 45,221 (95% CI: CAD 40,113–CAD 51,133) and CAD 28,599 (95% CI: CAD 24,965–CAD 32,824) for advanced and non-advanced DDLPS patients, respectively. For unresected and resected patients, the overall total mean cost PPY was CAD 41,186 (95% CI: CAD 36,727–CAD 46,474) and CAD 28,071 (95% CI: CAD 24,180–CAD 32,588), respectively. Cancer-related costs for specialist visits, inpatient hospitalizations, homecare visits, and cancer clinic visits were significantly higher for the advanced and unresected DDLPS groups.

## 4. Discussion

This study presents a comprehensive analysis of the epidemiology, treatment patterns, survival, HCRU, and costs associated with DDLPS in Ontario, Canada. The study findings highlight a higher incidence rate, similar baseline characteristics and treatment types, suboptimal survival rates, and a significant healthcare economic impact that is supported by the relevant literature.

The 684 incident cases and 0.465 per 100,000 overall incidence rate (2010–2022) in this study are higher than previously reported, with 182 incident cases of DDLPS (also in Ontario) from 1993 to 2015 [6,8] and 0.2 per 100,000 to 1 per 330,000 [12]. This increase in incidence could be attributable to increased awareness of the disease and improved diagnostic testing and availability of diagnostic codes. Furthermore, the cumulative prevalence rate of DDLPS in this study of 1.995 per 100,000 indicated that patients remained in the cohort until death or censorship, regardless of whether they experienced a recurrence. Nevertheless, a high recurrence rate among DDLPS patients was previously estimated to be 47% [13].

That the majority of DDLPS patients were male (64.3%), the mean age was 66.2 years, and 60.6% had a primary tumour site in the retroperitoneum or abdomen aligns with previous studies reporting DDLPS patients being 55.8–65% male [3,14], with a mean age of 63.6 years [14], and 59.5% having the retroperitoneum as primary tumour site [3]. Treatment-wise, 19.1% of DDLPS patients in this study received systemic (chemotherapy) treatment consistent with 16.5% in a previous US study [3]. Single-agent doxorubicin accounted for 61.5% of cases, which is consistent with current guidelines for managing patients with advanced or unresectable DDLPS [5]. In contrast, previous studies have reported that combination chemotherapy is the most commonly used first-line therapy (41–88%) [13,15]; however, these patient populations were heterogeneous based on disease type (WDLPS/DDLPS) [13] and only one tumour location (retroperitoneum only) [15].

Survival outcomes in the present study included an mOS of 69 (IQR = 15–121) months for all DDLPS patients, 60 (IQR = 14–151) months for retroperitoneum-located tumours, and 8 (IQR = 6–NA) months for those with stage IV disease. In a previous study conducted in the US, Gootee et al. (2018) reported comparable survival outcomes to our findings, with 63.6 months (5.3 years) for all DDLPS patients, 45.5 months for retroperitoneum-located tumours, and 10.2 months for stage IV disease [3]. On the other hand, we found that patients receiving 1L systemic treatment had an mOS of 16 (IQR = 6–58) months, which was lower compared to the mOS of 35 months reported by Gootee et al. (2018) [3]. Key points regarding survival include that younger DDLPS patients, those with non-retroperitoneal tumours, those diagnosed at an earlier stage, and patients who had their tumour resected within the first six months of diagnosis had significantly better survival rates, emphasizing the importance of early detection and surgical management if the patient is fit for resection and DDLPS is localized.

To the best of our knowledge, no prior study has described both all-cause HCRU and costs among patients with DDLPS. Two studies have examined medical costs associated with STS in Europe and in the US. One study reported a mean cost per STS patient of EUR 16,793 (2017) in the first two years of diagnosis, while the other reported a mean lifetime cost of EUR 65,616 (2015) per STS patient [10]. In our study, the mean all-cause healthcare cost among DDLPS patients was CAD 34,448 (95% CI: CAD 31,394–CAD 37,863) PPY for all years that increased to CAD 45,221 (95% CI: CAD 40,113–CAD 51,133) and CAD 41,186 (95% CI: CAD 36,727–CAD 46,474) for patients with advanced and unresected disease, respectively. Inpatient hospitalization was the largest cost driver for the overall DDLPS cohort, having the highest mean cost PPY of CAD 14,522, and this was similar to the mean cost of EUR 7,950 reported by Rugge et al. (2022) [14].

Study limitations include DDLPS being a rare disease and reliance on ICD-O-3 codes, which may have led to underdiagnosis and challenges in identifying tumour recurrence, as well as missing staging information for 48% of the DDLPS patients. For the latter, improving staging information in the future could help DDLPS patients receive appropriate treatment. The prevalence rate may be overestimated, as patients were counted from diagnosis until death or censoring. Use of 1L systemic treatment as a proxy for advanced (metastatic) cases, particularly by excluding untreated patients or those receiving unspecified treatments, could have led to selection bias. Treatment pattern reporting was limited by the exclusion of clinical trial drug data and the grouping of drug regimens due to small sample sizes. Additionally, treatment intent—such as whether the treatment was administered for curative or palliative purposes or adjuvant or neo-adjuvant purposes—could not be determined within any of the administrative databases that were used in the analysis. Lastly, medical oncologist visit costs were low despite the high number of therapeutic radiologist visits per person-year. This discrepancy could be explained by unbilled virtual care during COVID-19, as certain virtual care billing codes had no associated cost.

In conclusion, this is the first real-world evidence study to evaluate the epidemiology, clinical, and economic burden associated with DDLPS in Canada. DDLPS is a rare disease with limited treatment options; patients with advanced disease status have significantly lower survival rates and incur high HCRU and costs. Newer and more effective treatments (e.g., targeted therapies, immunotherapies) are needed for DDLPS patients to improve their survival. HCRU and cost information can inform decision-makers to ensure adequate resources are available to patients with this rare disease.

## Figures and Tables

**Table 1:** Baseline characteristics, treatment status, and follow-up time of total DDLPS cohort, stratified by advanced and resected status.

Baseline Characteristics	Total DDLPS Cohort	Advanced DDLPS			Unresected Status		
	N = 611	Yes N = 246	No N = 365	*p*-Value	Yes N = 373	No N = 238	*p*-Value
Age at Index Date, Years							
Mean (SD)	66.2 (13.3)	64.8 (12.8)	67.1 (13.6)	0.0408	67.0 (13.2)	64.8 (13.3)	0.0485
Median (IQR)	67 (57–76)	66 (56–73)	67 (57–77)	0.0573	68 (57–77)	66 (56–75)	0.0856
Min–max	21–96	21–91	26–96		26–96	21–93	
Age Group at Index Date (N, %)							
18–50 years	76 (12.4%)	34 (13.8%)	42 (11.5%)	0.1523	41 (11.0%)	35 (14.7%)	0.4137
51–64 years	186 (30.4%)	76 (30.9%)	110 (30.1%)		110 (29.5%)	76 (31.9%)	
65–74 years	173 (28.3%)	77 (31.3%)	96 (26.3%)		110 (29.5%)	63 (26.5%)	
75+ years	176 (28.8%)	59 (24.0%)	117 (32.1%)		112 (30.0%)	64 (26.9%)	
Sex (N, %)							
Female	218 (35.7%)	80 (32.5%)	138 (37.8%)	0.1809	136 (36.5%)	82 (34.5%)	0.6135
Male	393 (64.3%)	166 (67.5%)	227 (62.2%)		237 (63.5%)	156 (65.5%)	
Residence (N, %)							
Urban	531 (86.9%)	209 (85.0%)	322 (88.2%)	0.2414	318 (85.3%)	213 (89.5%)	0.1297
Rural	80 (13.1%)	37 (15.0%)	43 (11.8%)		55 (14.7%)	25 (10.5%)	
Treatment facility in one of five cancer centres *** (N, %)							
Yes	317 (51.9%)	133 (54.1%)	184 (50.4%)	0.3753	166 (44.5%)	151 (63.4%)	<0.0001
Type of Centre for Diagnosis (N, %)							
Community	207 (33.9%)	75 (30.5%)	132 (36.2%)	0.1460	147 (39.4%)	60 (25.2%)	0.0003
Academic	404 (66.1%)	171 (69.5%)	233 (63.8%)		226 (60.6%)	178 (74.8%)	
Income Quintile (N, %)							
1 (lowest)	104 (17.0%)	34 (13.8%)	70 (19.2%)	0.1139	61 (16.4%)	43 (18.1%)	0.9894
2-	119 (19.5%)	59 (24.0%)	60 (16.4%)		73 (19.6%)	46 (19.3%)	
3-	151 (24.7%)	56 (22.8%)	95 (26.0%)		93 (24.9%)	58 (24.4%)	
4-	115 (18.8%)	47 (19.1%)	68 (18.6%)		71 (19.0%)	44 (18.5%)	
5 (highest)	122 (20.0%)	50 (20.3%)	72 (19.7%)		75 (20.1%)	47 (19.7%)	
Charlson Co-Morbidity Index Score (N, %)							
Score = 0	419 (68.6%)	173 (70.3%)	246 (67.4%)	0.2855	251 (67.3%)	168 (70.6%)	0.1533
Score = 1	23 (3.8%)	11 (4.5%)	12 (3.3%)		15 (4.0%)	8 (3.4%)	
Score = 2	102 (16.7%)	42 (17.1%)	60 (16.4%)		58 (15.5%)	44 (18.5%)	
Score ≥ 3	67 (11.0%)	20 (8.1%)	47 (12.9%)		49 (13.1%)	18 (7.6%)	
Disease Stage (N, %)							
Stage I	70 (11.5%)	11 (4.5%)	59 (16.2%)	NA	** 38–42	** 28–32	0.0008
Stage II	85 (13.9%)	12 (4.9%)	73 (20.0%)		43 (11.5%)	42 (17.6%)	
Stage III	143 (23.4%)	143 (58.1%)	-		75 (20.1%)	68 (28.6%)	
Stage IV	20 (3.3%)	20 (8.1%)	-		** 15–19	* 1–5	
Unknown/missing	293 (48.0%)	60 (24.4%)	233 (63.8%)		198 (53.1%)	95 (39.9%)	
Topography (N, %)							
Retroperitoneum/ abdomen	370 (60.6%)	166 (67.5%)	204 (55.9%)	0.0578	257 (68.9%)	113 (47.5%)	<0.0001
Extremities	121 (19.8%)	42 (17.1%)	79 (21.6%)		27 (7.2%)	94 (39.5%)	
Thorax or trunk	40 (6.5%)	12 (4.9%)	28 (7.7%)		** 19–23	** 17–21	
Pelvis	54 (8.8%)	16 (6.5%)	38 (10.4%)		45 (12.1%)	9 (3.8%)	
Other or unknown	26 (4.3%)	10 (4.1%)	16 (4.4%)		** 21–25	* 1–5	
Years of Follow-up Time (from Index Date to Death or Censored Date)							
Mean (SD)	4.1 (3.5)	3.6 (3.3)	4.4 (3.6)	0.0027	3.3 (3.2)	5.4 (3.5)	<0.0001
Median (IQR)	3.1 (1.1–6.6)	2.5 (1.1–5.2)	3.6 (1.1–7.3)	0.0100	2.0 (0.6–5.3)	4.9 (2.2–8.2)	<0.0001
Treatment after DDLPS Diagnosis Anytime During Follow-up (N, %)							
Surgical status	321 (52.5%)	123 (50.0%)	198 (54.2%)	0.3026	83 (22.3%)	238 (100.0%)	<0.0001
Radiation status	398 (65.1%)	181 (73.6%)	217 (59.5%)	0.0003	226 (60.6%)	172 (72.3%)	0.0031
Systemic therapy (ALR/NDFP data only)	117 (19.1%)	117 (47.6%)	-	NA	85 (22.8%)	32 (13.4%)	0.0042

* 1–5 indicates small cell suppression as per ICES policy; other ** X–X number range is to prevent back calculation. *** Cancer centres include Mount Sinai Hospital, Princess Margaret Hospital/University Health Network, London Health Sciences Centre, Juravinski Cancer Centre, and Ottawa Hospital Cancer Centre.

**Table 2:** Incidence and prevalence information of DDLPS patients in Ontario, Canada.

	Crude Incidence Rate per 100,000	Crude Prevalence Rate per 100,000
Overall cohort (2010–2022)	0.46	1.99
Age		
18–50	0.10	0.56
51–64	0.58	2.81
65–74	1.15	4.86
75+	1.56	5.17
Sex		
Female	0.32	1.31
Male	0.62	2.72

**Table 3:** Treatment information for DDLPS patients.

Line of Treatment	Drug Type	Frequency (%)
1L treatment (N = 117)	DOXO	95 (81.2)
	GEM	16 (13.7)
	OTHER	6 (5.1)
Mean time from DDLPS diagnosis to 1L treatment in days (SD) Median time from DDLPS diagnosis to 1L treatment in days (IQR)	624.7 (862.1) 222 (70–899)	
Mean time spent on 1L treatment in days (SD) Median time spent on 1L treatment in days (IQR)	85.3 (123.2) 50 (22–113)	
Mean time from 1L to 2L (SD) Median time from 1L to 2L (IQR)	156.4 (292.4) 28 (21–87)	
2L treatment (N = 41)	GEM	22 (53.7)
	OTHER or DOXO *	19 (46.3)
Mean time spent on 2L treatment in days (SD) Median time spent on 2L treatment in days (IQR)	84.8 (121.4) 45 (22–90)	

Abbreviations: 1L = first-line; 2L = second-line; DOXO = doxorubicin; GEM = gemcitabine; OTHER = not DOXO or GEM, includes carboplatin and paclitaxel with radiotherapy, dacarbazine, epirubicin and ifosfamide, paclitaxel (weekly), pazopanib, vincristine, dactinomycin, and cyclophosphamide, ifosfamide, olaratumab (maintenance), paclitaxel and carboplatin, durvalumab. * DOXO was included with other in 2L to avoid small-cell suppression, since less than 5 patients received DOXO as 2L.

**Table 4:** Survival probabilities at year 1, 5, and 10, and median overall survival of DDLPS.

Variable	Details	No. (%) Patients at Index	1-Year Survival % (95% CI)	5-Year Survival % (95% CI)	10-Year Survival % (95% CI)	Median OS Months (IQR)
Overall cohort	All DDLPS patients	611 (100.0%)	76.6 (73.2–80.0)	52.4 (48.2–56.7)	35.0 (29.7–40.3)	69 (15–151)
Age	<65 years	262 (42.9%)	83.2 (78.7–87.7)	67.3 (61.3–73.3)	51.5 (43.5–59.4)	122 (28–NA)
	≥65 years	349 (57.1%)	71.6 (66.9–76.4)	41.2 (35.6–46.8)	21.7 (15.0–28.5)	47 (11–114)
Topography	Retroperitoneum/abdomen	370 (60.6%)	75.7 (71.3–80.0)	48.5 (43.0–54.1)	31.4 (24.9–38.0)	60 (14–151)
	Other	241 (39.4%)	78.0 (72.8–83.2)	58.3 (51.7–64.9)	40.1 (31.4–48.9)	101 (17–NA)
Disease Stage	Stage I	70 (11.5%)	90.0 (83.0–97.0)	64.2 (51.8–76.6)	* 1–5	85 (43–NA)
	Stage II	85 (13.9%)	85.9 (78.5–93.3)	64.3 (54.1–74.6)	46.0 (33.7–58.4)	103 (30–NA)
	Stage III	143 (23.4%)	78.3 (71.6–85.1)	55.4 (45.9–64.8)	34.5 (21.4–47.6)	79 (19–145)
	Stage IV	20 (3.3%)	45.0 (23.2–66.8)	* 1–5	0.0%	8 (6–NA)
	Unknown/missing	293 (48.0%)	72.0 (66.9–77.2)	48.0 (41.9–54.1)	31.3 (23.9–38.7)	60 (12–134)
Advanced DDLPS	Yes	246 (40.3%)	76.4 (71.1–81.7)	43.7 (36.8–50.6)	21.9 (14.7–29.1)	53 (15–108)
	No	365 (59.7%)	76.7 (72.4–81.0)	57.9 (52.6–63.3)	43.6 (36.5–50.6)	103 (16–NA)
Resected Status	Resected	238 (39.0%)	91.2 (87.6–94.8)	73.2 (67.1–79.3)	52.1 (43.1–61.1)	131 (57–NA)
	Not resected	373 (61.0%)	67.3 (62.5–72.1)	39.1 (33.8–44.5)	23.8 (17.5–30.0)	38 (9–115)
1L Drug Treatment	All patients with 1L treatment (based on drug group)	117				16 (6–58)
2L Drug Treatment	All patients with 2L treatment (based on drug group)	41				9 (5–16)

* 1–5 indicates small-cell suppression as per ICES policy; † Median survival months from start date of 1L drug treatment to death or censoring date; median survival months from start date of 2L drug treatment to death or censoring date. NA indicates not available. This occurs due to either (1) a small sample size and insufficient data separation to calculate the upper end of the interquartile range (IQR) for the median survival value, or (2) the patient group not reaching the 25% survival rate (or 75% mortality rate) within the study period.

**Table 5:** All-cause healthcare resource utilization for overall DDLPS patients (all years and year 1).

Healthcare Resource Utilization	Total No. Patients Who Used Resource	Mean HCRU Use per Person-Year (95% CI)	
		All years	Year 1 **
Specialist visits	611 (100%)	22.1 (20.2–23.7)	47.6 (44.7–50.5)
Medical oncologist visits	295 (48.3%)	2.5 (1.9–3.1)	3.6 (2.9–4.4)
Therapeutic radiologist visits	506 (82.8%)	1.8 (1.7–2.0)	6.1 (5.6–6.7)
Inpatient hospitalizations	584 (95.6%)	0.8 (0.8–0.9)	2.2 (2.0–2.4)
Length of stay (days)	584 (95.6%)	8.9 (7.8–10.2)	27.1 (23.6–30.9)
Homecare visits	502 (82.2%)	19.0 (15.6–22.9)	29.5 (25.5–33.8)
Cancer clinic visits	453 (74.1%)	5.2 (4.7–5.6)	17.8 (16.5–19.0)

** Year 1 post-DDLPS diagnosis.

**Table 6:** All-cause healthcare resource utilization for DDLPS patients by advanced and non-advanced status for all years.

	Advanced Status N = 246		Non-Advanced Status N = 365		*p*-Value
Healthcare Resource Utilization	Total No. (%) Patients Who Used HCRU	Mean HCRU Use PPY (95% CI)	Total No. (%) Patients Who Used HCRU	Mean HCRU Use PPY (95% CI)	
Specialist visits	246 (100.0%)	30.1 (27.2–33.2)	365 (100.0%)	17.7 (16.2–19.5)	<0.0001
Medical oncologist visits	159 (64.6%)	5.9 (4.5–7.4)	136 (37.3%)	0.6 (0.4–0.9)	<0.0001
Therapeutic radiologist visits	213 (86.6%)	2.29 (2.0–2.6)	293 (80.3%)	1.6 (1.4–1.8)	0.0411
Inpatient hospitalizations	* 241–245	1.1 (1.0–1.3)	* 339–343	0.7 (0.6–0.8)	0.0002
Length of stay (days)	* 241–245	11.3 (9.4–13.5)	* 339–343	7.7 (6.4–9.2)	0.1432
Homecare visits	216 (87.8%)	25.0 (19.1–32.1)	286 (78.4%)	15.8 (11.7–20.3)	0.2018
Cancer clinic visits	218 (88.6%)	7.4 (6.6–8.4)	235 (64.4%)	4.0 (3.6–4.4)	<0.0001

* X–X number range is to prevent back calculation.

**Table 7:** All-cause healthcare costs (2023 CAD) for overall DDLPS patients for all years and year 1 post-DDLPS diagnosis.

Healthcare Resource Utilization	Total No. Patients Who Used Resource	Mean Cost per Person-Year (95% CI)	
		All years	Year 1 **
Total costs	611 (100%)	CAD 34,448 (CAD 31,394–CAD 37,863)	CAD 86,840 (CAD 80,120–CAD 94,236)
Specialist visits	611 (100%)	CAD 4895 (CAD 4564–CAD 5249)	CAD 12,566 (CAD 11,756–CAD 13,379)
Medical oncologist visits	295 (48.3%)	CAD 201 (CAD 159–CAD 251)	CAD 336 (CAD 273–CAD 406)
Therapeutic radiologist visits	506 (82.8%)	CAD 334 (CAD 305–CAD 365)	CAD 1137 (CAD 1053–CAD 1216)
Inpatient hospitalizations	584 (95.6%)	CAD 14,522 (CAD 12,804–CAD 16,505)	CAD 43,373 (CAD 37,757–CAD 49,882)
Homecare visits	502 (82.2%)	CAD 2122 (CAD 1796–CAD 2486)	CAD 3921 (CAD 3409–CAD 4492)
Cancer clinic visits	453 (74.1%)	CAD 4314 (CAD 3860–CAD 4801)	CAD 12,345 (CAD 11,378–CAD 13,298)

** Year 1 post-DDLPS diagnosis.

**Table 8:** All-cause healthcare costs stratified by advanced and resected status for all years.

Mean Cost PPY (95% CI)	Advanced Status			Unresected Status		
	Yes (N = 246)	No (N = 365)	*p*-Value	Yes (N = 373)	No (N = 238)	*p*-Value
Total cost	CAD 45,221 (CAD 40,113–CAD 51,133)	CAD 28,599 (CAD 24,965–CAD 32,824)	<0.05	CAD 41,186 (CAD 36,727–CAD 46,474)	CAD 28,0701 (CAD 24,180–CAD 32,588)	<0.05
Specialist visits	CAD 6260.4 (CAD 5645–CAD 6940)	CAD 4154 (CAD 3783–CAD 4560)	<0.05	CAD 5718 (CAD 5194–CAD 6298)	CAD 4116 (CAD 3727–CAD 4543)	<0.05
Medical oncologist visits	CAD 465 (CAD 357–CAD 587)	CAD 58 (CAD 40–CAD 80)	<0.05	CAD 303 (CAD 228–CAD 393)	CAD 105 (CAD 74–CAD 141)	<0.05
Therapeutic radiologist visits	CAD 447 (CAD 392–CAD 510)	CAD 273 (CAD 241–CAD 307)	<0.05	CAD 398 (CAD 352–CAD 449)	CAD 274 (CAD 240–CAD 313)	<0.05
Inpatient hospitalization	CAD 18,779 (CAD 15,601–CAD 22,804)	CAD 12,202 (CAD 10,264–CAD 14,456)	<0.05	CAD 18,954 (CAD 16,168–CAD 22,257)	CAD 10,319 (CAD 8440–CAD 12,666)	<0.05
Homecare visits	CAD 2839 (CAD 2290–CAD 3482)	CAD 1732 (CAD 1346–CAD 2165)	<0.05	CAD 2721 (CAD 2207–CAD 3290)	CAD 1555 (CAD 1160–CAD 2009)	<0.05
Cancer clinic visits	CAD 7477 (CAD 6462–CAD 8591)	CAD 2590 (CAD 2264–CAD 2947)	<0.05	CAD 5226 (CAD 4485–CAD 6066)	CAD 3450 (CAD 2965–CAD 3982)	<0.05

## Data Availability

The dataset from this study is held securely in coded form at ICES. While legal data sharing agreements between ICES and data providers (e.g., healthcare organizations and government) prohibit ICES from making the dataset publicly available, access may be granted to those who meet pre-specified criteria for confidential access, available at www.ices.on.ca/DAS (email: das@ices.on.ca). The full dataset creation plan and underlying analytic code are available from the authors upon request, understanding that the computer programmes may rely upon coding templates or macros that are unique to ICES and are therefore either inaccessible or may require modification.

## Supplementary Materials

The following supporting information can be downloaded at: https://www.mdpi.com/article/10.3390/curroncol32050273/s1, Figure S1: Flow chart of study cohort (684 cohort for incidence/prevalence, 611 for other analyses); Figure S2: Incidence and prevalence of DDLPS from 2010 to 2022; Table S1: Unadjusted and adjusted cox regression models to determine factors associated with survival in patients with DDLPS.
