# Supplementary material for: Epidemiology, Treatment Patterns, Survival, Healthcare Resource Utilization, and Costs of Dedifferentiated Liposarcoma (DDLPS) in Canada: A Retrospective Cohort Study Using Administrative Databases in Ontario
Source: Curr Oncol. 2025 May 9;32(5):273. doi: 10.3390/curroncol32050273 (PMC12109657; doi:10.3390/curroncol32050273)
Supplement: Supplementary file 1 [file curroncol-32-00273-s001.zip › curroncol-3584974-supplementary.pdf]

Supplemental Figure S1. Flow chart of study cohort (684 cohort for incidence/prevalence, 611 for other analyses)

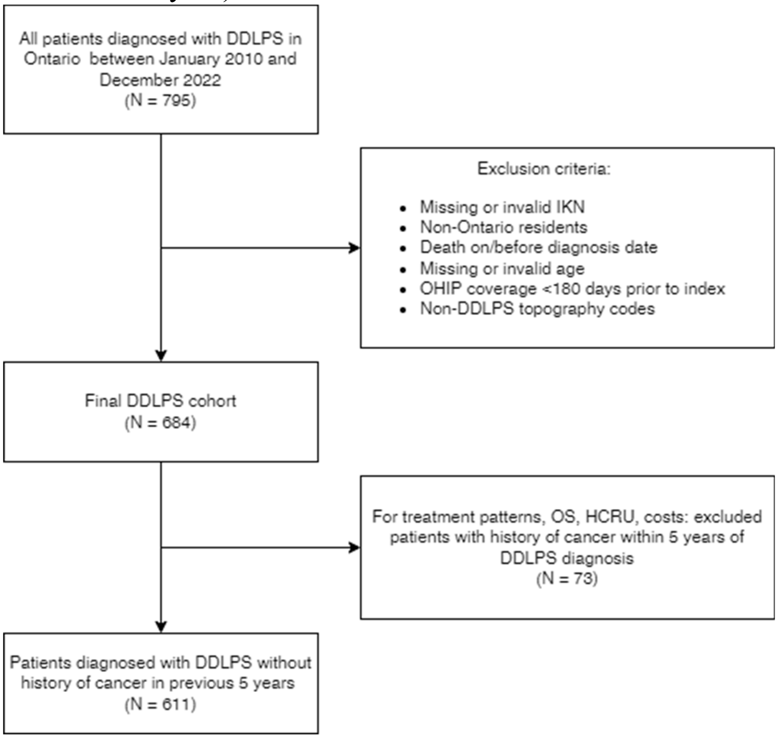

Supplemental Figure S2. Incidence and prevalence of DDLPS from 2010 to 2022

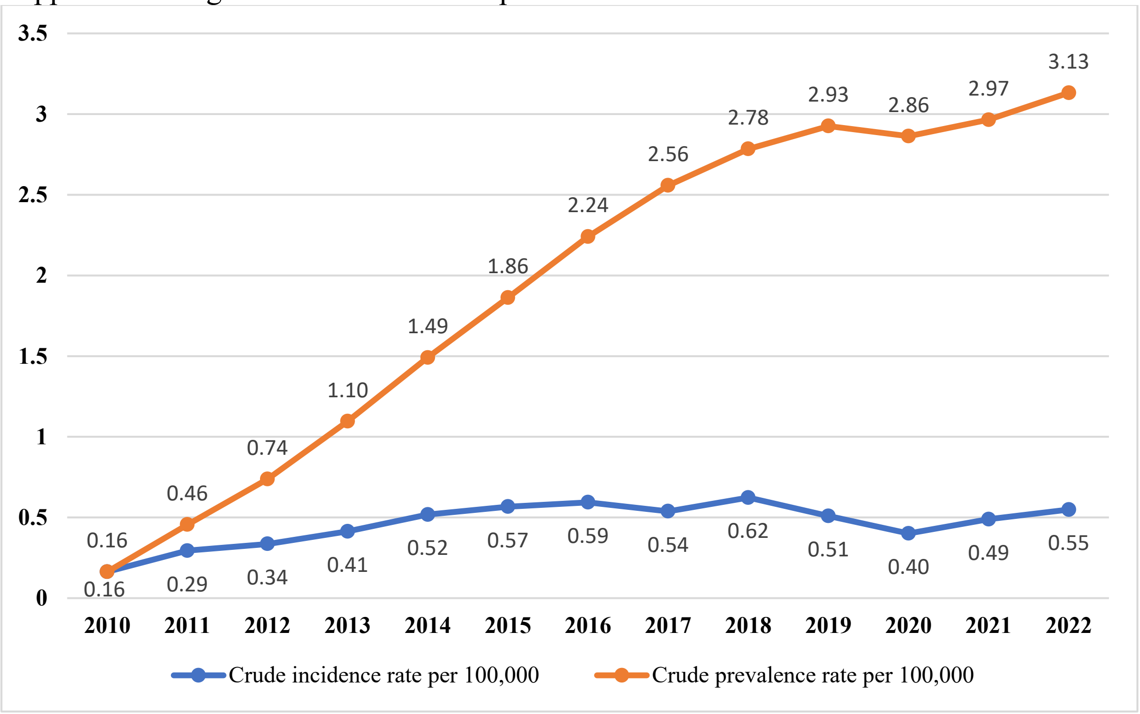

Supplemental Table S1. Unadjusted and adjusted cox regression models to determine factors associated with survival in patients with DDLPS

Variable	Unadjusted HR (95% CI)	Adjusted HR (95% CI)
Age (65+ vs. <65)	2.05 (1.62-2.59)	2.10 (1.66-2.67)
Sex (Male vs. Female)	0.84 (0.67-1.06)	0.91 (0.72-1.14)
Primary tumor site (Retro/Abdomen vs. Other)	1.30 (1.03-1.63)	1.33 (1.06-1.67)
Disease stage		
Stage: II vs. I	1.05 (0.65-1.71)	1.12 (0.69-1.82)
Stage: III vs. I	1.40 (0.90-2.20)	1.34 (0.86-2.11)
Stage: IV vs. I	6.22 (3.47-11.14)	6.80 (3.79-12.21)
Stage: Missing vs. I	1.76 (1.17-2.65)	1.68 (1.12-2.53)
